# Supplementary material for: Identification of a Novel SSTR3 Full Agonist for the Treatment of Nonfunctioning Pituitary Adenomas
Source: Cancers (Basel). 2023 Jun 30;15(13):3453. doi: 10.3390/cancers15133453 (PMC10340464; doi:10.3390/cancers15133453)
Supplement: Supplementary file 1 [file cancers-15-03453-s001.zip › cancers-2429507-supplementary.pdf]

Supplementary Materials: Identification of a Novel SSTR3 Full Agonist for the Treatment of Non-Functioning Pituitary Adenomas

Daniela Modena, Maria Luisa Moras, Giovanni Sandrone, Andrea Stevenazzi, Barbara Vergani, Pooja Dasgupta, Andrea Kliewer, Sebastian Gulde, Alessandro Marangelo, Mathias Schillmaier, Raul M. Luque, Stephen Bäuerle, Natalia S. Pellegata, Stefan Schulz and Christian Steinkühler

1. Synthesis of ITF2984[1]

1.1. General procedure for the Solid phase amino acid coupling

Fmoc protected amino acid (4 eq.) was dissolved in DMF. HBTU (4 eq.), HOBt (4 eq.) and DIPEA (8 eq.) were added. The reaction mixture was then added to the resin (1 eq.) and stirred at r.t. for 2 hours. The resin was filtered and washed with DMF and DCM.

1.2. Cyclo[Tyr(Bn)-Phe-Pro(4-OCONH(CH2)2NH2)-Tyr-3,8-diMeONal-Lys] (ITF2984)

The linear hexapeptide was obtained by solid phase synthesis starting from the resin Fmoc-Lys(Boc)-Trt(Cl)-DVB. The amino acids were added in the following order: Fmoc-3,8-diMeONal-OH, Fmoc-Tyr(tBu)-OH, Fmoc-Hyp-OH, Fmoc-Phe-OH, Fmoc-Tyr(Bzl)-OH. For every cycle, the Fmoc protective group was removed by treating the resin with 20% piperidine in DMF for 30 minutes and the subsequent Fmoc-protected amino acid was activated and coupled to the amino groups available on the resin as described above in the general procedure. After the 3rd cycle, a solution of *p*-nitrophenylchloroformate (5 eq.) and *N*-methylmorpholine (5 eq.) in DCM was added to the resin. The reaction mixture was stirred for 2.5 h, then filtered and washed with DCM. A solution of tert-butyl (2-aminoethyl)carbamate in DCM was added to the resin. After 2h the resin was filtered and washed before carrying out the subsequent coupling cycles.

After the last Fmoc-deprotection step, the resin was treated with a mild-cleavage solution of TFE/AcOH/DCM 2:1:7 for 30 minutes at room temperature. The solvents were removed under reduced pressure and the residue was dissolved in EtOAc and washed with a 5% $NaHCO_3$ solution. The solvents were removed under reduced pressure.

The protected linear peptide obtained was dissolved in dry DMF and the solution was cooled at -10°C. DIPEA (2 eq.) and diphenylphosphorylazide (1.3 eq.) were added. The reaction mixture was stirred at 0°C for 60h. DMF was removed under reduced pressure and the residue was dissolved in EtOAc and washed with a 5% $NaHCO_3$ solution and brine. The organic phase was dried over Na_2SO_4, filtered, and evaporated to dryness.

The cyclic peptide obtained was treated with a solution of 30% TFA in water at 0°C for 30 minutes. The two isomers obtained were separated and purified by prep-HPLC (HPLC/MS system with Waters Symmetry C_{18} 5mm 19x50 mm column, equipped with Waters ZQ mass spectrometer) in order to obtain the pure product (95.4%; m/z = 1149 amu [M+H]+ and 575 [M+2H]2+).

2. Radioligand binding to human SSTR

2.1. Assay procedure

The binding affinity of ITF2984 for human SSTR subtypes was determined in competitive radioligand binding assay using cell membranes of CHO-K1 cell line transfected with DNA coding sequence corresponding to human SSTR1, SSTR2, SSTR3, SSTR5 proteins, respectively GeneBank protein sequence NP_001040.1, NP_001041.1, NP_001042.1, NP_001043.1.

The reaction mixture was prepared as follows:

- 50 µl of assay buffer (60 µl for total binding determination) 25 mM Hepes pH 7.4, 5 mM $MgCl_2$, 1 mM $CaCl_2$, 10 µg/ml Saponin, 0.5% protease free BSA.

- 10 μl of SST agonist at increasing concentrations (Serial dilutions from master solution in 100% DMSO)
- 20 μl of membrane extracts (SSTR1 - 2μg; SSTR2 - 0.2μg; SSTR3 - 0.7μg; SSTR5 - 0.7μg)
- 20 μl of radioligand (3-[^{125}I] iodotyrosyl^{11} Somatostatin-14 Amersham, IM161, 2000 Ci/mmol) (Radioligand final concentration: SSTR1 – 0.11nM; SSTR2 - 0.50nM; SSTR3 - 0.07nM; SSTR5 - 0.13nM)

Somatostatin-28 (Bachem, H-4955) was used as reference compound.

After 60 min at 25°C in a water bath, samples were filtered over GF/B filters (Perkin Elmer, 6005177) presoaked in 0.5% PEI for 2h at RT) with a Filtermate Harvester (Perkin Elmer). Filters were washed 6 times with 0.5 ml of ice-cold filtration buffer, incubated 15 min. on an orbital shaker with 50 μl of Microscint 20 (Packard) and counted for radioactivity with a TopCount™ or MicroBeta™ for 1 min/well.

Dose-response curve were performed for each SSTR (agonist range 10^{-11} – $3x10^{-7}$ M), Each point was performed in duplicate[2,3]

2.2. Data analysis

Dose-response data from test compounds were analysed with XLfit (IDBS) software using nonlinear regression applied to a sigmoidal dose-response model.

Agonist activity of test compounds is expressed as a percentage of the activity of the reference agonist at its EC_{100} concentration.

2.3. Inhibition of GHRH-induced GH release in rat pituitary primary cell culture

Methods for isolation of rat pituitary cells and *in vitro* GH production were adapted from previously described procedures.[4–6]

2.4. Setting-up of rat pituitary primary culture

Animal procedures and ethical revisions were performed according to the current Italian legislation (Legislative Decree March 4, 2014, n. 26), enforcing the 2010/63/UE Directive on the protection of animals used for biomedical research. Rat pituitary glands (Sprague-Dowley - Harlan) were minced, treated with collagenase Type IV (Sigma C5138) 1 mg/ml, passed first through 5 ml syringe and finally through a 40 μm mesh filter to obtain a single cell suspension.

Cells were counted in a Burker's chamber in the presence of Trypan blue to determine cell viability. Number of pituitary cells was about 0.8-$1x10^{6}$ cells/rat and viability was > 90%.

Cells were resuspended in 100 ml of complete medium (DMEM containing 5% fetal calf serum, 5% horse serum, 20 mM Hepes pH 7, 1% NEAA, Pen/Strep) and plated 150000 cells/ml/well in 48 wells plate. 750 μl of medium were changed after 3-4 days and primary cultures used for experiment after 6-7 days.

2.5. GHRH-induced GH release

Plates were carefully washed once with Hank's solution and further incubated with 1 ml Hank's containing 0.1% BSA, 20 mM HEPES for 1 h, to decrease background.

Medium was removed and replaced with fresh 240 μl Hank's, 0.1% BSA, 20 mM HEPES. 30 μl of $3x10^{-8}$ M in H_2O rat GHRH (10x solution) (Growth Hormone Releasing Hormone Sigma G6646) and 30 μl of testing material (10x solution) were added sequentially. In the above conditions GHRH was $3x10^{-9}$ M final conc. Testing compounds were prepared 10x concentrated in a range 10^{-5} -10^{-9} M (final conc 10^{-6}-10^{-10} M). Test was run in duplicate. Plates were incubated at 37°C for 3 h. After incubation, supernatants were collected and frozen immediately at -20°C until assay for GH determination.

2.6. GH assay

GH produced by primary cultures was tested by ELISA assay using Rat Growth Hormone Biotrak Enzyme immunoassay Mouse/Rat Growth ELISA (DSL-10-72100) or Rat/Mouse Growth Hormone ELISA (Millipore EZRMGH-45K) diluting supernatants 1:20, according to manufacturer's instructions.

3. SSTR internalization

3.1. HEK293 cell line

HEK293 cells were obtained from the German Resource Centre for Biological Material (DSMZ, Braunschweig, Germany) and grown in Dulbecco's modified Eagle's medium supplemented with 10% fetal calf serum in a humidified atmosphere containing 5% CO_2. Cells were transfected with plasmid encoding murine HA-tagged SSTR2, SSTR3 or SSTR5 receptors using Lipofectamine according to the instructions of the manufacturer (Invitrogen, Carlsbad, CA). Stable transfectants were selected in the presence of 500 µg/ml G-418. Stable cells were characterized using radioligand-binding assays, Western blot analysis, surface ELISA assay and immunocytochemistry as described previously.[7–9]

HEK293 cells stably expressing HA-tagged human somatostatin receptors cells were seeded onto poly-L-lysine coated 24-well plates overnight. On the next day, cells were pre-incubated with anti-HA antibody for 2 h at 4°C. Cells were then transferred to 37°C, exposed to 1 µM agonist for 30 min at 37°C and fixed with 4 % paraformaldehyde and 0.2 % picric acid in phosphate buffer (pH 6.9) for 30 minutes at room temperature (RT). After washing the coverslips with PBS w/o Ca^{2+}/Mg^{2+} buffer several times, cells were blocked with phosphate buffer containing 3% NGS for 2 hours and were then incubated with Alexa488-conjugated secondary antibody (1:2,000) (LifeTechnologies, Thermo Fisher Scientific A11008) over night at 4°C. On the next day, cells were washed several times with PBS w/o Ca^{2+}/Mg^{2+} and specimens were mounted with Roti®-MountFluorCare DAPI (Carl Roth, HP20.1) and examined using a Zeiss LSM510 META laser scanning confocal microscope (Zeiss, Jena, Germany).

3.2. U2OS human cell lines transfected with human receptors (SSTR2-tGFP, SSTR3-tGFP, SSTR5-tGFP).

Compounds were tested at five different concentrations (10^{-5}, 10^{-6}, 10^{-7}, 10^{-8}, 10^{-9} M) in comparison to untreated cells. 10^{-6} M SST28 (Sigma-Aldrich S6135) was included as positive control.

U2OS recombinant cell lines were obtained from Innoprot (Derio, Spain). Cells were thawed and maintained in DMEM-F12 medium supplemented with 10% FCS at 37ºC in a humidified atmosphere with 5% CO_2. After 24 hours, cells were detached using Trypsin-EDTA solution, centrifuged, plated $1x10^4$ cells per well in 96-well plates and incubated as above for further 24 h. For SST agonist testing, the culture media was removed, and the test compounds were added to the cells in OptiMeM culture medium (Life technologies 51985-034) for 3 h (SSTR2, SSTR3) or 7 h (SSTR5). Test was performed in triplicate. The receptor internalization was quantified prior to formaldehyde fixation (3.7% wt., 20 minutes). Nuclei were stained using DAPI (2 µg/ml) and the fluorescence was measured using a BD Pathway 855 High-Content Bioimager from Becton Dickinson. The receptor internalization was calculated using AttoVision Software. Approximately 500 cells per field were analyzed.

Both Excel 2003 and Sigmaplot 9.0 were used for data management.

Agonist activity of test compounds was calculated relatively to positive control (SST28 10^{-6} M) and showed as a percentage of activity.

4. SSTR phosphorylation.

4.1. Western blot analysis

Western blot analysis was performed as previously described.[7–9] Briefly, HEK293 cells stably expressing the human somatostatin receptors were seeded onto poly-L-lysine-coated 60 mm dishes and grown to 90 % confluency. After agonist-stimulation, cells were lysed in RIPA buffer (50 mM Tris-HCl, pH 7.4, 150 mM NaCl, 5 mM EDTA, 1 % Nonidet P-40, 0,5 % sodium deoxycholate, 0,1 % SDS) containing protease and phosphatase inhibitors (Complete mini and PhosSTOP; Roche Diagnostics, Mannheim, Germany). Pierce™ HA epitope tag antibody beads (Thermo Scientific, Rockford, USA) were used to enrich HA-tagged somatostatin receptors or wheat germ lectin-agarose beads for no-tagged somatostatin receptors. To elute proteins from the beads, the samples were incubated in SDS sample buffer for 20 min at 60°C. Supernatants were separated from the beads, loaded on 8% SDS polyacrylamide gels and immunoblotted onto nitrocellulose membranes afterwards. After blocking, membranes were incubated with phosphorylation state-specific somatostatin receptor antibodies: pS337/pT341-SST3 (7TM0357A), pS341/pS343-SST2 (7TM0356A), pT333-SST5 (7TM0359A) (all obtained from 7TM Antibodies GmbH, Jena, Germany) at a dilution of 1:1000 at 4°C overnight. Membranes were incubated in HRP-linked secondary antibody for 2 hours, followed by detection using a chemiluminescence system (90 mM p-coumaric-acid, 250 mM luminol, 30% hydrogen peroxide). Blots were subsequently stripped and reprobed with the following phosphorylation-independent somatostatin receptor antibodies non-phospho-SST3 (7TM0357N), non-phospho-SST2 (7TM0356N), non-phospho-SST5 (7TM0359N) or anti-HA antibodies (7TM000HA) (all obtained from 7TM Antibodies GmbH, Jena, Germany) to confirm equal loading of the gel. Protein bands on Western blots were exposed to X-ray films.

4.2. Membrane potential assay

HEK293 cells were stably transfected with either HA-tagged SSTR2 or SSTR3 or SSTR5 and GFP conjugated GIRK2 channel plasmids (Origene). Cells were seeded in 96-well plates and allowed to grow at 37°C and 5% CO_2 for 48 hours. Hank's Balanced Salt Solution (HBSS) buffered with 20 mM HEPES solution (1.3 mM $CaCl_2$, 5.4 mM KCl, 0.4 mM K_2HPO_4, 0.5mM $MgCl_2$, 0.4 mM $MgSO_4$, 136.9 mM NaCl, 0.3 mM Na_2HPO_4, 4.2 mM $NaHCO_3$ and 5.5 mM glucose; pH 7.4) was used to wash the cells. The membrane potential dye (FLIPR Membrane Potential kit BLUE, Molecular Devices) was reconstituted according to the manufacturer's instructions. To each well, 90 µl of the HBSS/HEPES buffer solution and an equal volume of the membrane potential dye was added, making the initial volume 180 µl per well and cells were incubated at 37°C for 45 minutes. Test compounds were prepared in buffer solution containing HBSS and 20 mM HEPES solution (pH 7.4) at 10 times the final concentration to be measured. Fluorescence measurements were performed in a FlexStation 3 microplate reader (Molecular Devices) at 37°C with excitation at 530 nm and emission at 565 nm. Baseline readings were taken every 1.8 s for 1 min. After 60 s, a volume of 20 µl of the test or vehicle control was injected into each well containing cells incubated with dye, to give a final in-well volume of 200 µl which resulted in a 1:10 dilution of the test compound. The change in fluorescence of the dye was recorded for 240 s using SoftMax Pro software.[10,11]

Table S1. Backbone torsion values related to (L,D Trp)-Lys dyad detected in several complexes between SSTR2, Somatostatin-14, Octreotide and Lanreotide. .

**Structure (pdb code) SSTR2-SRIF-14**	$\phi_{(i+1)}$	$\psi_{(i+1)}$	$\phi_{(i+2)}$	$\psi_{(i+2)}$	**Turn(s) type**
7T10	53.17	-128.9	-102.99	14.97	≈ β II′
7Y27	53.74	-130.2	-103.8	10.78	≈ β II′
7WJ5	59.77	-134.3	-102.7	11.26	≈ β II′

7XMR	-118.6	-61.23	-144.7	3.08	3 out of 4 β II′
7XAT	-55.32	-69.57	-137.3	24.08	**≈ β I**
7WIC	-55.14	108.8	47.15	31.94	**≈ β II**
Structure (pdb code) SSTR2-Octreotide					
7Y26	67.8	-111.3	-121.8	21.5	≈ β II′
7Y24	80.6	-120.2	-115.9	-8.0	≈ β II′
7XAU	75.00	-118.3	-115.4	15.4	≈ β II′
7T11	72.0	-106.7	-135.8	18.2	≈ β II′
Structure (pdb code) SSTR2-Lanreotide					
	88.61	-100.6	-138.8	-14.9	≈ β II′

Table S2. Backbone torsion values related to **X**-Lys dyad detected in several complexes between SSTR2, Somatostatin-14, Octreotide and Lanreotide. **X**= Trp, D-Trp, 3,5 di-MethOxy-D-2-Naphtylalanine for SRIF-14, Octreotide/Pasireotide and ITF2984, respectively).

Ligand – X residue	$\phi_{(i+1)}$	$\psi_{(i+1)}$	$\phi_{(i+2)}$	$\psi_{(i+2)}$	Turn(s) type
**<u>SSTR2</u>**					
SRIF14-Trp	22 (15)	-110 (27)	-88 (20)	15 (13)	≈β II′
Pasireotide D-Trp	-82(13)	90 (17)	70 (21)	21(47)	≈β II
ITF2984 – (3,5 diMeO-D-2Nal)	109 (123)	-148 (9)	-88 (11)	-37 (12)	≈β II′/ β I
Octreotide D-Trp	87 (11)	-113 (9)	-144 (33)	145 (10)	≈β II′
**<u>SSTR3</u>**					
SRIF14-Trp	-141 (27)	-112 (27)	-104 (24)	-4 (14)	≈β I
Pasireotide D-Trp	-69 (9)	75 (13)	61 (15)	5 (25)	β II
ITF2984 – (3,5 diMeO-D-2Nal)	143 (73)	-146 (17)	-79 (12)	-39 (11)	≈β II′/β I
Octreotide D-Trp	95 (14)	-118 (13)	-126 (13)	2 (18)	β II′

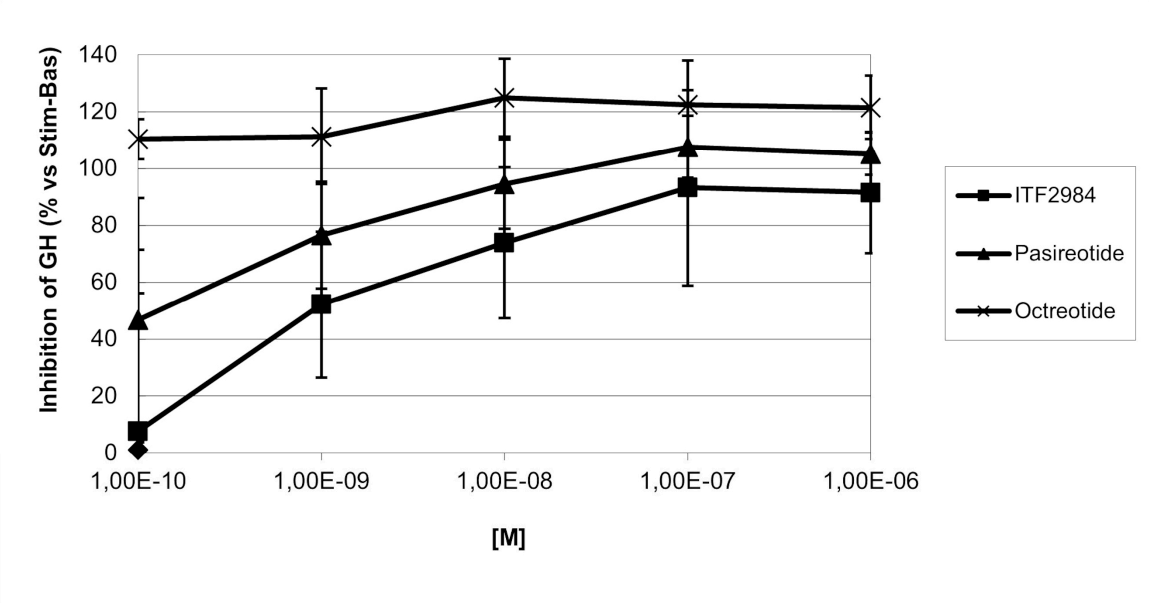

Figure S1. Inhibition of GHRH-stimulated GH release in vitro using primary cultures of rat anterior pituitary cells. ITF2984, Pasireotide and Octreotide have been tested in the concentration range 10^{-6} -10^{-10} M. GH released in the supernatant by primary cultures was determined by ELISA assay. Results are expressed as mean values ± SD of 3 experiments.

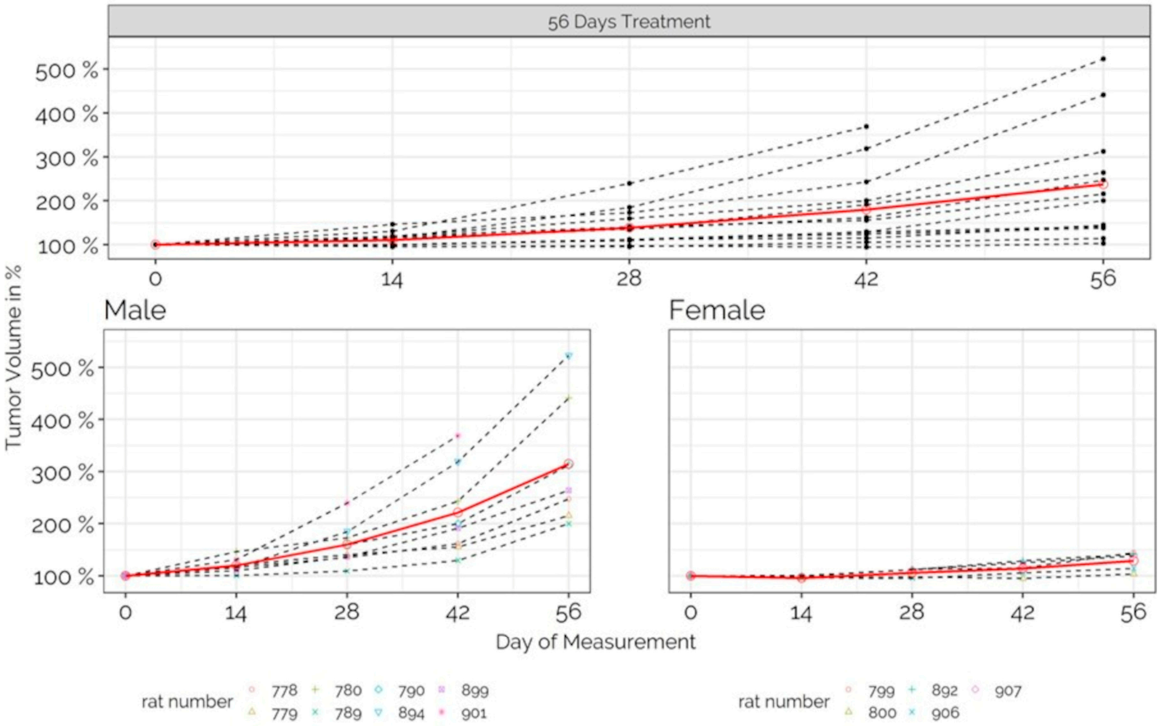

Figure S2. Trace plots of relative tumor volume in 56d ITF2984-treated group. Upper plot: Trace plots of relative tumor volume for 7.5-month-old mutant ITF2984-treated male and female rats together. Reported is the tumor volume relative to volume at day 0 of each rat. Bottom plots: Left, subpopulation of male rats. Right, female rats. Linear mixed effects (LME) models were applied for longitudinal analysis of tumor volume growth with absolute tumor volume at day 0 used for scaling subsequent measurements of each individual as previously reported. Relative volumes were transformed by natural logarithms for use as model outcomes in order to meet the normal distributional assumptions. Linear and quadratic growth predictors and interactions were considered for significance testing, performed by the F-test, with results presented as the mean ± standard error of the mean (SEM). Statistical significance between two series of data was determined by one-way ANOVA. A P value <0.05 was considered statistically significant.

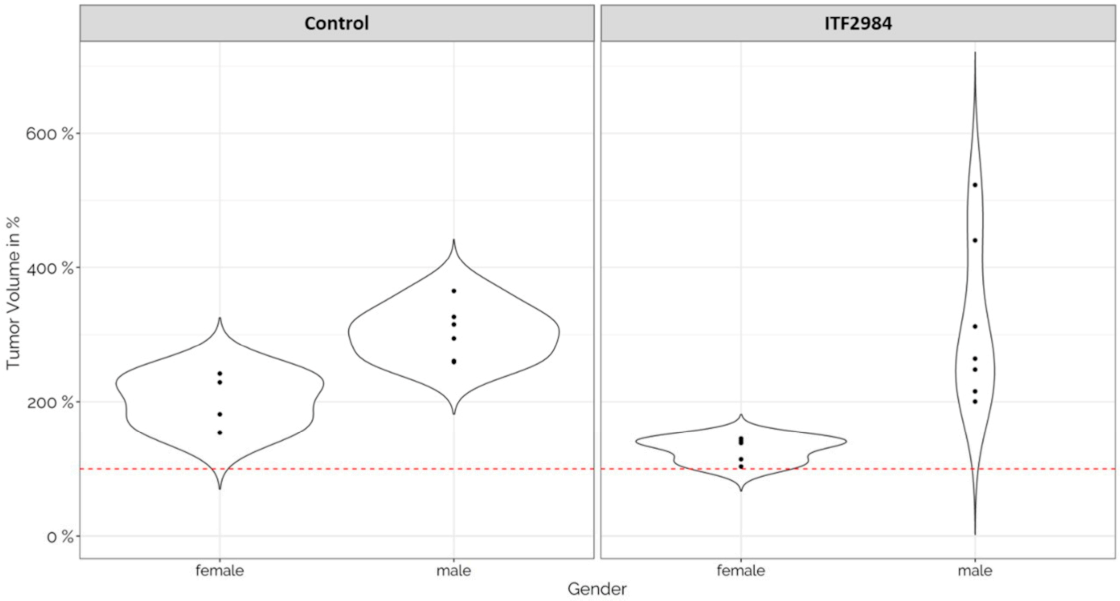

Figure S3. Distribution of relative tumor volume at end of the experiment. Distribution of relative tumor volume in 7.5-month-old rats treated with ITF2984 or placebo for 56 d in percentage when compared to baseline at day 0 (= 100 % tumor volume). Values were grouped by treatment and

gender. The red dashed line indicates 100% (no change in tumor volume). The distribution is shown as a violin plot.

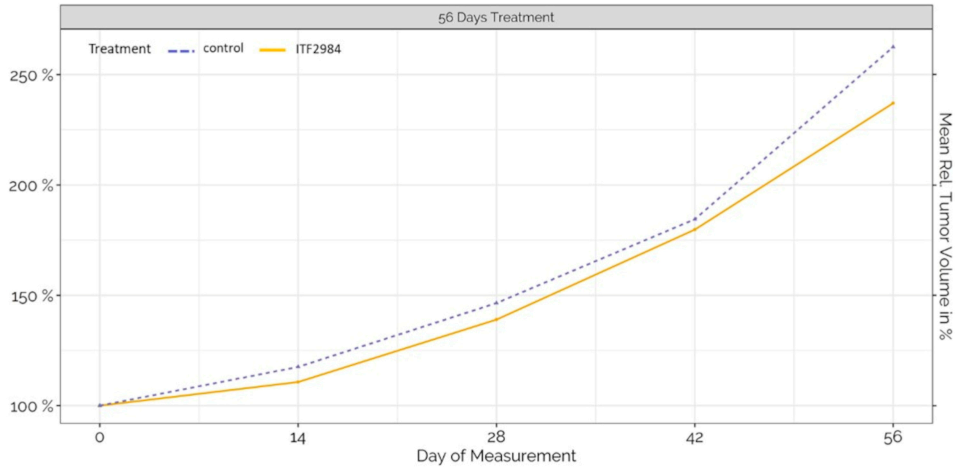

Figure S4. Mean relative tumor volume changes during treatment. Mean value slope of relative tumor volume for mutant rats of the two treatment groups and of both genders combined.

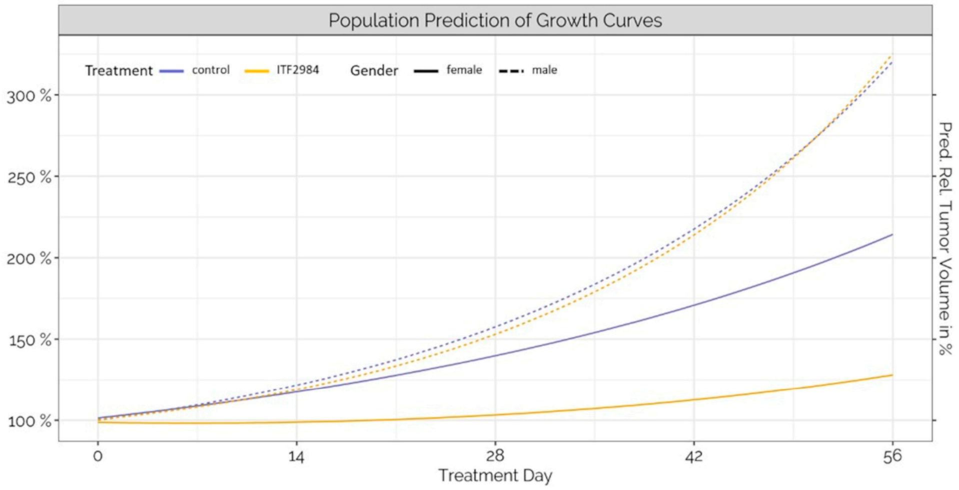

Figure S5. Predicted relative tumor volume changes during treatment. For each group the best fitting model was used to predict the group average rel. tumor volume (response) for each day and gender. Genders are shown separately.

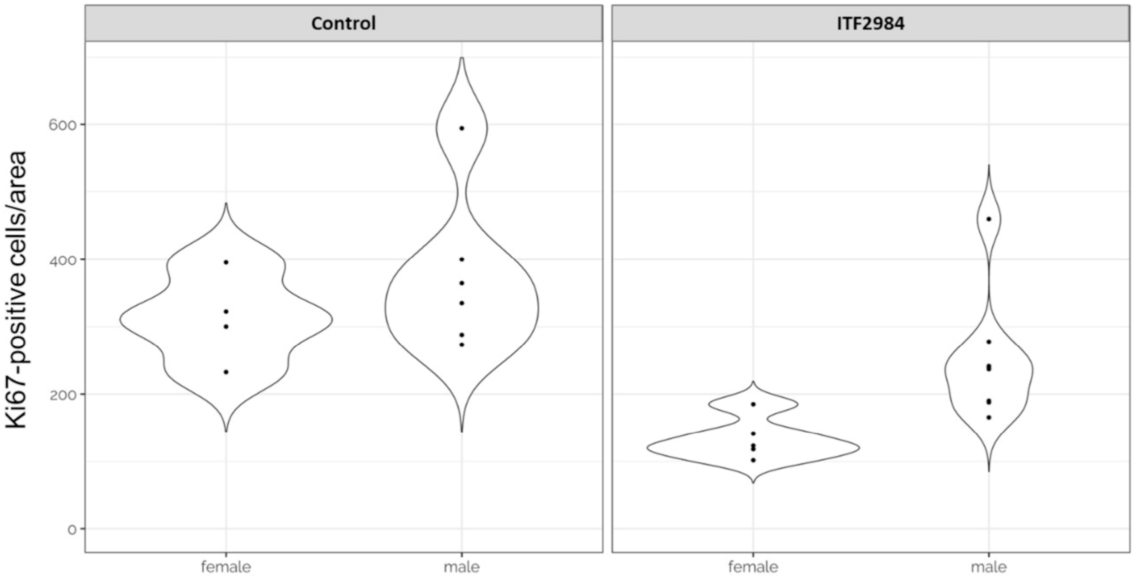

Figure S6. Proliferation of NF-PiNETs in placebo-treated (control) or ITF2984-treated rats. Number of Ki67-positive cells per 100.000 mm^2 in PitNETs of rats belonging to the 2 treatment groups and the 2 genders. The distribution is shown as a violin plot.

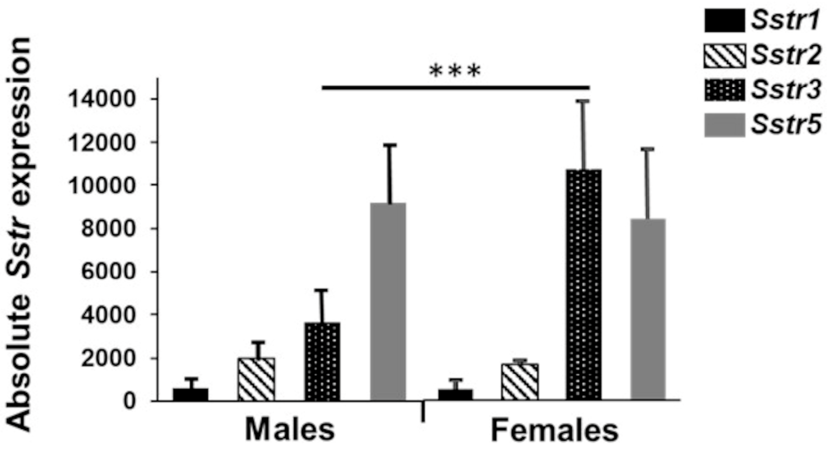

Figure S7. Expression of *Sstr* genes in MENX mutant rats. Absolute quantification of mRNA copy number/cell for *Sstr1,2,3,5* genes in placebo-treated control rats of both genders. Shown is the average ± SEM. *** p-value<0.0001.

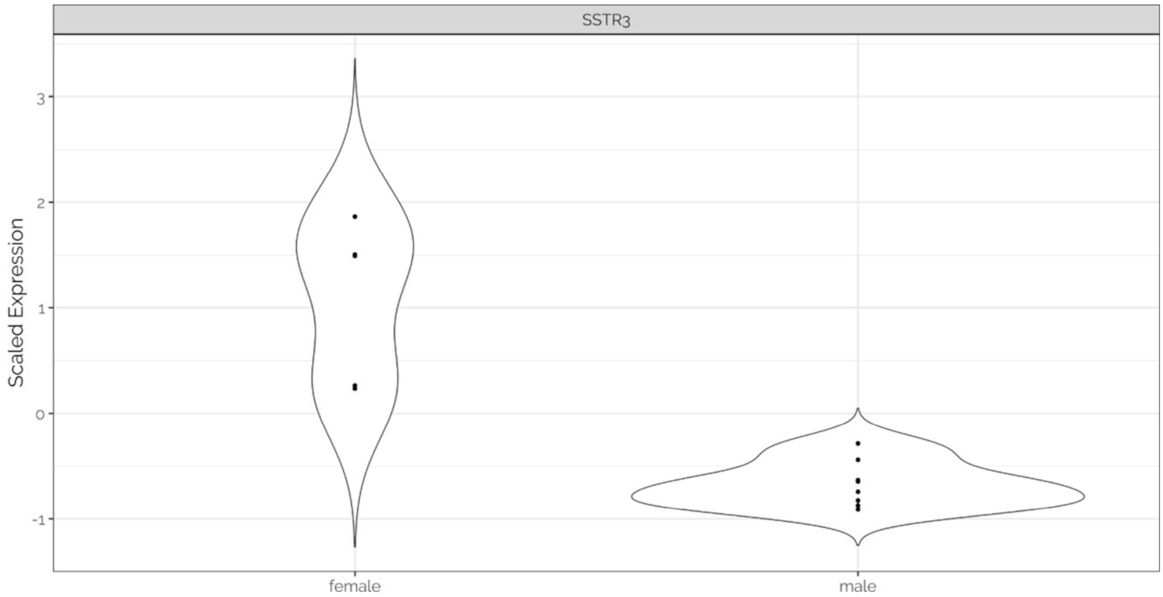

Figure S8. Expression of *Sstr3* at the end of ITF2984 treatment. Absolute quantification of mRNA copy number/cell for the *Sstr3* gene in tumors of rats of both genders following treatment with ITF2984 for 56d. *Sstr3* measurements are scaled by subtracting its mean value and dividing by its standard deviation independent of gender.

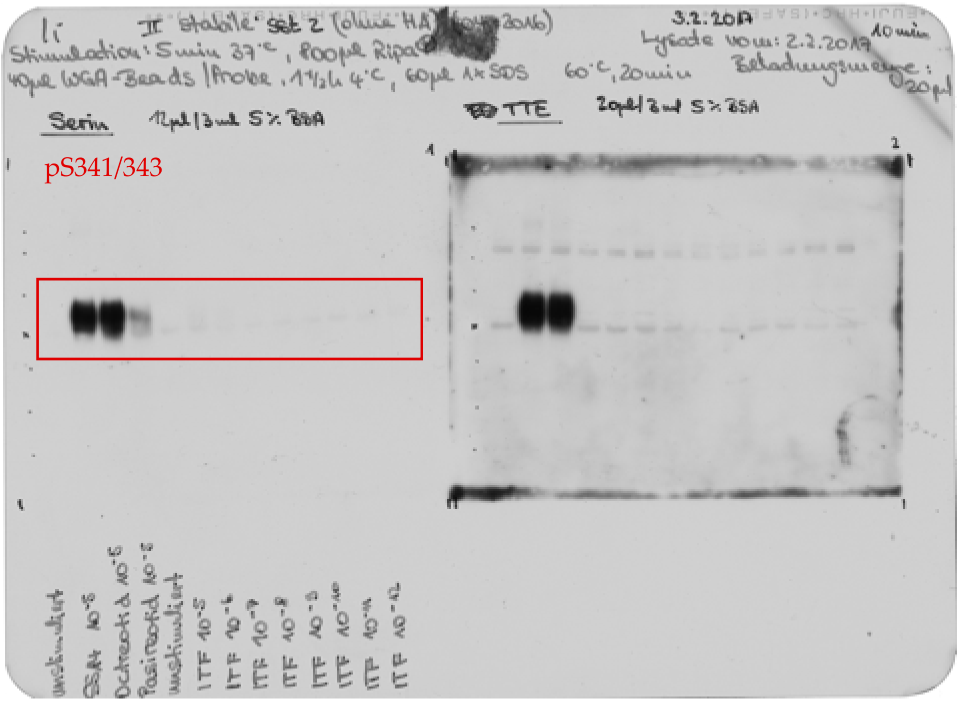

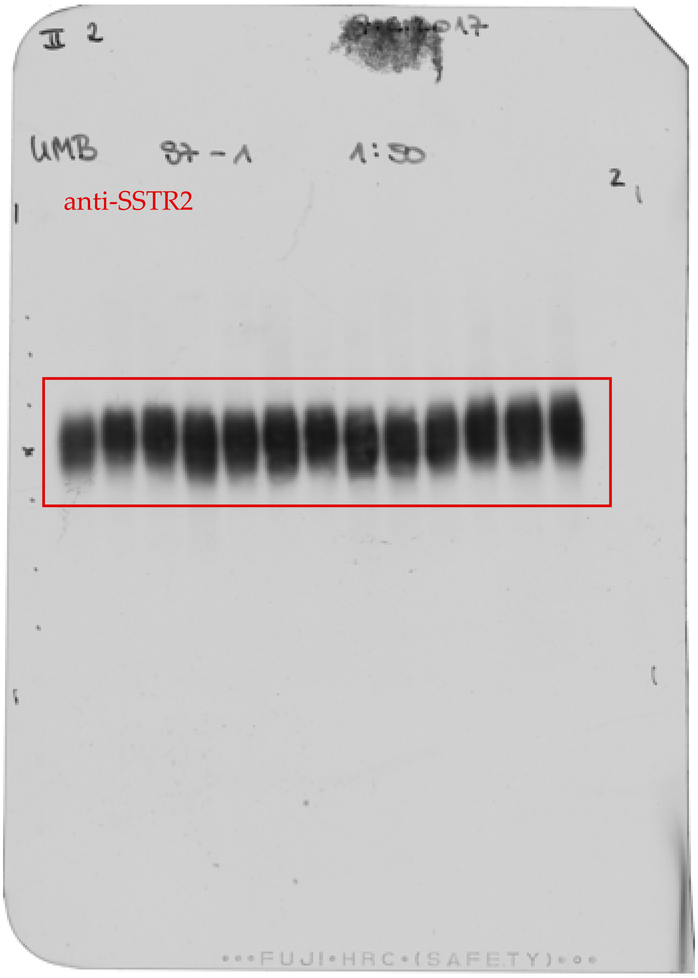

Figure S9. Western Blot SSTR2.

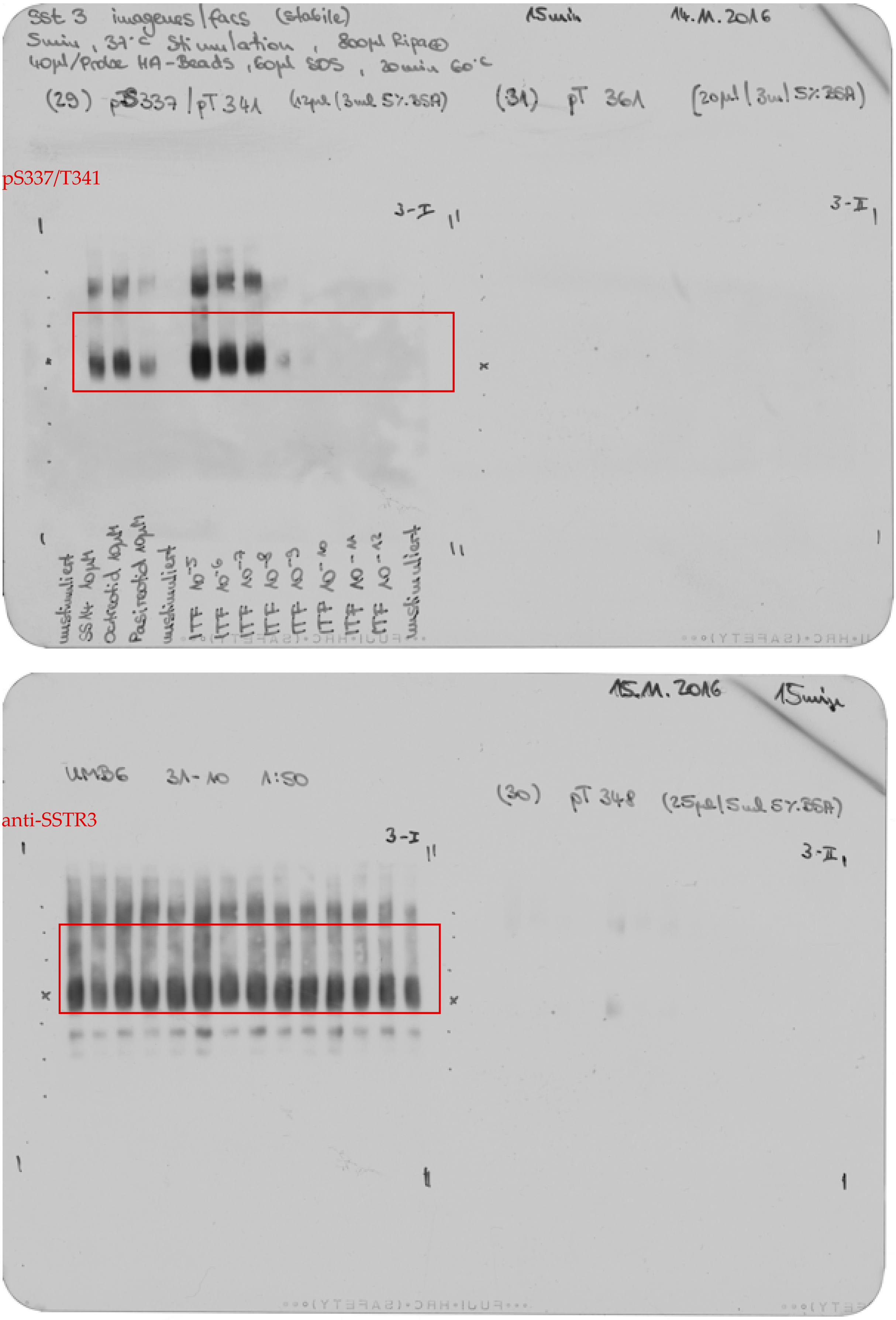

Figure S10. Western Blot SSTR3.

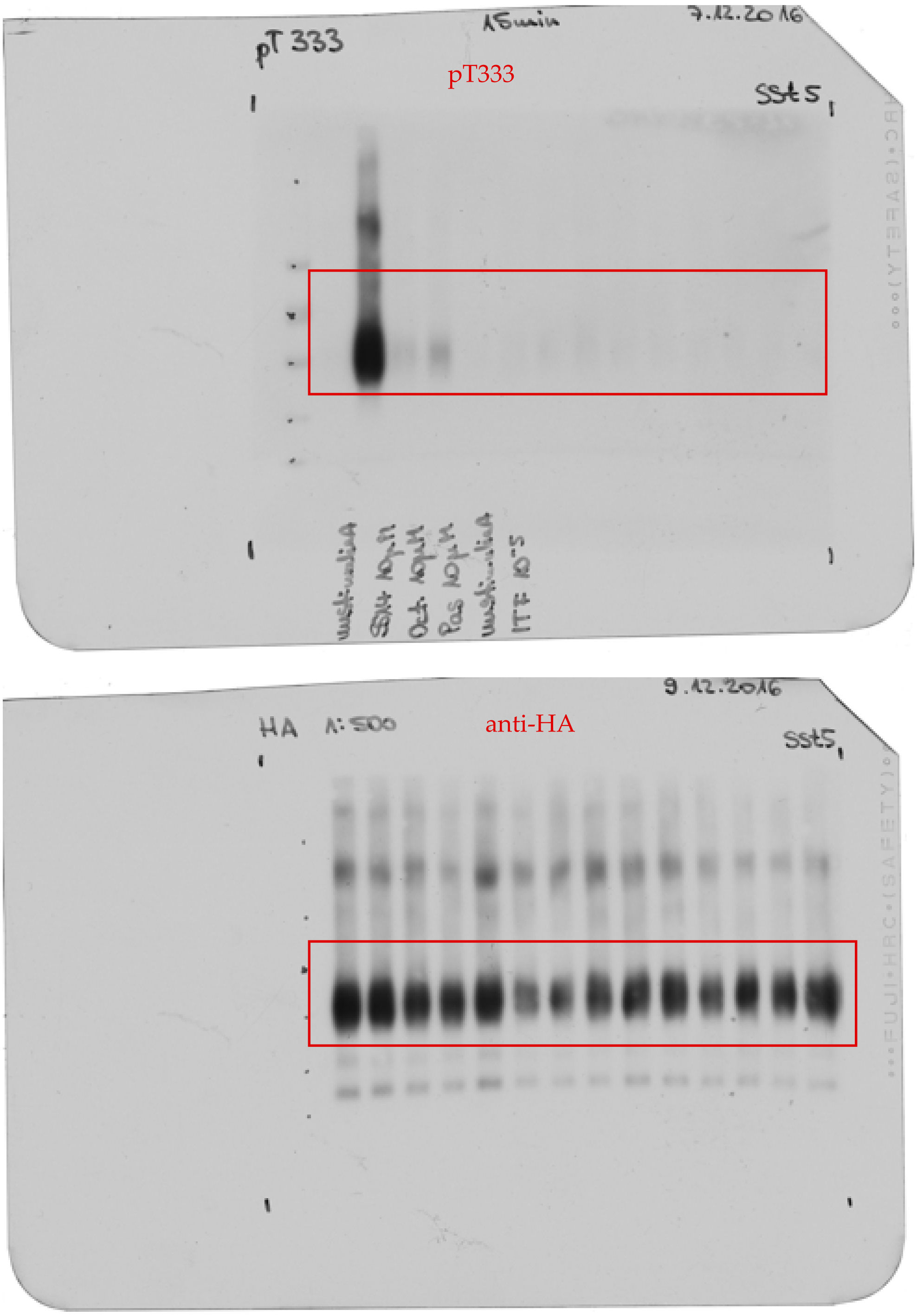

Figure S11. Western Blot SSTR5.

References

(1) Vitalli Andrea (IT), Pinori Massimo (IT), Mascagni Paolo (IT), (IT). New Non-Selective Somatostatin Analogues - WO2009071460, 2009.

(2) Bruns, C.; Raulf, F.; Hoyer, D.; Schloos, J.; Lübbert, H.; Weckbecker, G. Binding Properties of Somatostatin Receptor Subtypes. *Metabolism.* **1996**, *45* (SUPPL.1), 17–20, DOI: 10.1016/S0026-0495(96)90072-4.

(3) Siehler, S.; Seuwen, K.; Hoyer, D. Characterisation of Human Recombinant Somatostatin Receptors. 1. Radioligand Binding Studies. *Naunyn. Schmiedebergs. Arch. Pharmacol.* **1999**, *360* (5), 488–499, DOI: 10.1007/s002109900141.

(4) Vale, B. W.; Grant, G.; Burgess, C. In Vitro Pituitary Hormone Secretion Assay for Hypophysiotropic Substances Assay Method. **1970**, *61*, 82–93.

(5) Bruns, C.; Lewis, I.; Briner, U.; Meno-Tetang, G.; Weckbecker, G. SOM230: A Novel Somatostatin Peptidomimetic with Broad Somatotropin Release Inhibiting Factor (SRIF) Receptor Binding and a Unique Antisecretory Profile. *Eur. J. Endocrinol.* **2002**, *146* (5), 707–716, DOI: 10.1530/eje.0.1460707.

(6) Hofland, L. J.; Van Der Hoek, J.; Van Koetsveld, P. M.; De Herder, W. W.; Waaijers, M.; Sprij-Mooij, D.; Bruns, C.; Weckbecker, G.; Feelders, R.; Van Der Lely, A. J.; Beckers, A.; Lamberts, S. W. J. The Novel Somatostatin Analog SOM230 Is a Potent Inhibitor of Hormone Release by Growth Hormone- and Prolactin-Secreting Pituitary Adenomas in Vitro. *J. Clin. Endocrinol. Metab.* **2004**, *89* (4), 1577–1585, DOI: 10.1210/jc.2003-031344.

(7) Lehmann, A.; Kliewer, A.; Günther, T.; Nagel, F.; Schulz, S. Identification of Phosphorylation Sites Regulating Sst3 Somatostatin Receptor Trafficking. *Mol. Endocrinol.* **2016**, *30* (6), 645–659, DOI: 10.1210/me.2015-1244.

(8) Pöll, F.; Lehmann, D.; Illing, S.; Ginj, M.; Jacobs, S.; Lupp, A.; Stumm, R.; Schulz, S. Pasireotide and Octreotide Stimulate Distinct Patterns of Sst2A Somatostatin Receptor Phosphorylation. *Mol. Endocrinol.* **2010**, *24* (2), 436–446, DOI: 10.1210/me.2009-0315.

(9) Petrich, A.; Mann, A.; Kliewer, A.; Nagel, F.; Strigli, A.; Märtens, J. C.; Pöll, F.; Schulz, S. Phosphorylation of Threonine 333 Regulates Trafficking of the Human Sst5 Somatostatin Receptor. *Mol. Endocrinol.* **2013**, *27* (4), 671–682, DOI: 10.1210/me.2012-1329.

(10) Dasgupta, P.; Günther, T.; Reinscheid, R. K.; Zaveri, N. T.; Schulz, S. Rapid Assessment of G Protein Signaling of Four Opioid Receptors Using a Real-Time Fluorescence-Based Membrane Potential Assay. *Eur. J. Pharmacol.* **2021**, *890* (March 2020), 173640, DOI: 10.1016/j.ejphar.2020.173640.

(11) Günther, T.; Culler, M.; Schulz, S. Research Resource: Real-Time Analysis of Somatostatin and Dopamine Receptor Signaling in Pituitary Cells Using a Fluorescence-Based Membrane Potential Assay. *Mol. Endocrinol.* **2016**, *30* (4), 479–490, DOI: 10.1210/me.2015-1241.

Disclaimer/Publisher's Note: The statements, opinions and data contained in all publications are solely those of the individual author(s) and contributor(s) and not of MDPI and/or the editor(s). MDPI and/or the editor(s) disclaim responsibility for any injury to people or property resulting from any ideas, methods, instructions or products referred to in the content.
